# Oncocytic Hyperplastic Nodule Versus Oncocytic Adenoma: Diagnostic Controversies Through a Brief Investigative Case Series Study

**DOI:** 10.7759/cureus.60361

**Published:** 2024-05-15

**Authors:** Christos Topalidis, Georgios Petrakis, Triantafyllia Koletsa

**Affiliations:** 1 Department of Pathology, School of Medicine, Aristotle University of Thessaloniki, Thessaloniki, GRC

**Keywords:** molecular diagnostics, thyroid nodule, thyroid neoplasm, oncocytes, oncocytic adenoma

## Abstract

Oncocytes are frequently encountered in routine thyroidectomies. The distinction between oncocytic hyperplastic nodules and oncocytic adenomas (OAs) may be challenging. Although both entities are benign, a precise diagnosis is essential. We present two cases of solitary oncocytic lesions carrying pathogenic mutations in the p53 and NRAS genes, respectively, leading to a histological diagnosis of oncocytic hyperplastic nodules. Additionally, similar oncocytic nodules from two cases of autoimmune thyroiditis did not show any significant findings on molecular analysis (next-generation sequencing, NGS). Hence, this brief investigative series study is of particular diagnostic interest because it prompts pathologists to use the term adenoma when a solitary oncocytic nodule is encountered, regardless of the established criteria for the diagnosis of adenoma. This viewpoint leads to the possible need for the reevaluation of the histological criteria of adenomas when it comes to oncocytic lesions in order to gain a common diagnostic approach and nomenclature among pathologists and overcome any controversies in such cases.

## Introduction

Oncocyte derives from the Greek word “onkos,” meaning swelling or mass. It is an enlarged, polygonal cell with distinct cell borders and abundant granular, eosinophilic cytoplasm due to the accumulation of mitochondria and prominent nucleoli [1]. Thyroid tumors in which at least 75% of the cells have the appearance of oncocytes are designated as oncocytic [2]. The use of the term “Hürthle” should be avoided since it is a misnomer. Instead, the terms “oncocyte” and “oncocytic” are accurate, and therefore, they should be preferred [3,4].

Oncocytes are a common finding in thyroidectomy specimens. Pathologists can encounter them either under the setting of reactive processes such as nodular hyperplasia (as hyperplastic nodule) and Hashimoto’s disease or as part of a neoplastic process, namely oncocytic adenoma (OA), oncocytic carcinoma (OCA), oncocytic variant of papillary thyroid carcinoma (PTC), or oncocytic variant of medullary carcinoma [4, 5].

According to several reports, OAs concern 10-15% of thyroid nodules with a preceding indeterminate fine-needle aspiration (FNA) diagnosis [4]. Moreover, in the largest multi-international study that has been carried out so far, oncocytic predominant nodules constitute 1.8% of the total FNA samples, and most of the histologically benign cases proved to be OAs (28.5%) [6].

The presence of oncocytes in thyroidectomy surgical specimens may raise differential diagnostic problems. To reach the correct diagnosis, pathologists take into account their nuclear characteristics, overall architecture, and benign or infiltrative nature. One diagnostic challenge is the distinction between oncocytic hyperplastic nodules and oncocytic adenomas.

Accurate diagnosis of oncocytic lesions is essential, especially when taking into account that they have been linked with more aggressive behavior [7]. Moreover, we must not forget that there are several reports in the literature of allegedly benign oncocytic nodules giving rise to metastasis [7,8]. The aim of this brief investigative study is to highlight the differential diagnostic challenges and controversies arising from oncocytic nodules through the histological, immunohistochemical, and molecular study of four cases.

## Case presentation

Our four patients underwent a total thyroidectomy with all the relevant presurgical clinical examinations and blood work-ups needed. Clinically, they presented with symptoms related to multinodular goiter without any clinical or laboratory findings or meaningful differences between them to report.

Case 1

**Figure 1:**
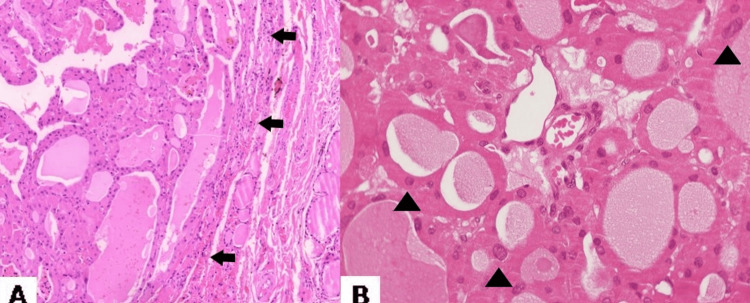
(A) HE ×40: Solitary oncocytic nodule surrounded by an ambiguous thin capsule (arrows) without signs of compression of the neighboring thyroid tissue. (B) HE ×400: The nodule comprises of oncocytes (arrowheads) with distinct cell borders, abundant eosinophilic cytoplasm and prominent nucleoli.

The initial stimulus was the case of a woman in her early 40s who underwent a total thyroidectomy due to nodular hyperplasia. A thyroidectomy specimen weighing 27 g was sent to our Pathology Department. Serial sections through the entire gland revealed a white-tan nodule in the lower pole of the right lobe, with the greatest diameter of 0.8 cm. Haematoxylin and eosin (H&E)-stained sections showed that the nodule consisted entirely of oncocytes arranged in follicles of various sizes and shapes, a few of which showed dilatation. There were cystic degeneration areas and a few pseudopapillary structures (Figure 1A). The oncocytes showed no significant pleomorphism or atypia (Figure 1B). There were no nuclear features in the PTC. The nodule was circumscribed and partially surrounded by a fine fibrous capsule without evidence of compression of the neighboring follicles (Figure 1A). The rest of the gland had a nodular configuration with nodules of various sizes, admixed with areas of hemorrhage, fibrosis, mild lymphocytic infiltration, follicular rupture, and clusters of macrophages. Despite the multiple sections, oncocytes were not found in any other area of the thyroid gland.

The above findings could lead to the diagnosis of either an oncocytic hyperplastic nodule or an oncocytic adenoma. The absence of compression of the surrounding follicles favors the hypothesis of an oncocytic hyperplastic nodule. However, the solitary oncocyte-filled nodule in a single area raised concerns about a clonal process. Next-generation sequencing (NGS) analysis (Illumina platform) was followed and revealed a pathogenic p53 mutation in exon 8, supporting the diagnosis of oncocytic adenoma.

Case 2

**Figure 2:**
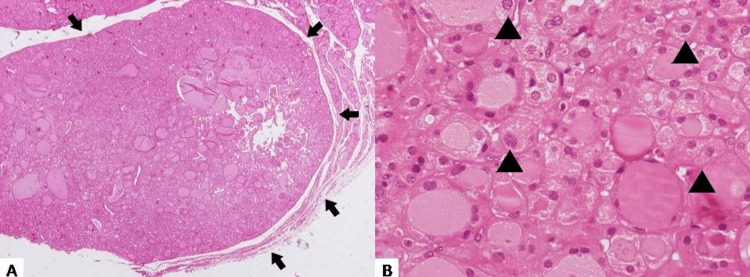
(A) HE ×40 and (B) HE ×400: Same features were identified in a second solitary oncocytic nodule. This nodule also harbored a pathogenic mutation as the previous (arrows pinpoint the ambiguous capsule of the nodule, while the arrowheads highlight the typical features of oncocytes).

The above finding intrigued us enough to investigate the profile of molecular alterations in oncocytes in three other recently diagnosed cases with the presence of oncocytic nodules. We retrieved a similar case with a solitary oncocytic nodule (Figure 2A-2B) that had previously been diagnosed as an oncocytic hyperplastic nodule in the setting of nodular hyperplasia without presenting oncocytic cells in any other area of the thyroid specimen.

Cases 3 and 4

**Figure 3:**
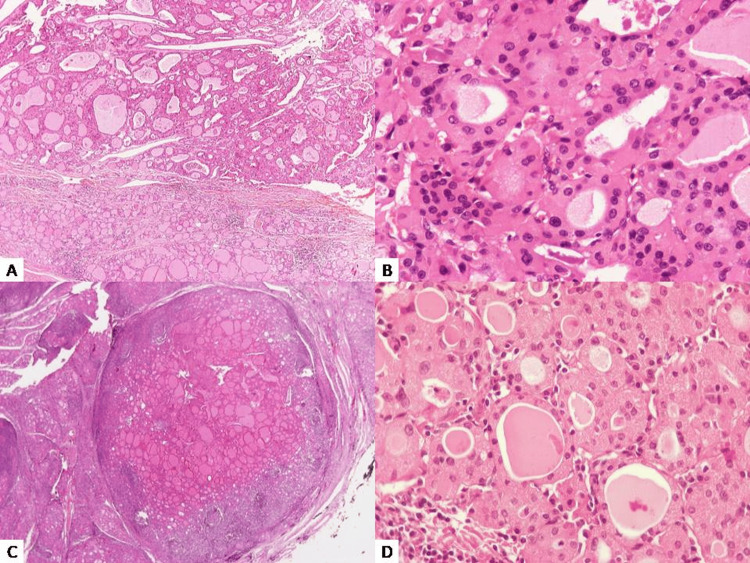
(A) HE ×40, (B) HE ×400 and (C) HE ×40, (D) HE ×400: Two solitary oncocytic nodules in the setting of Hashimoto disease (A, B & C, D, respectively). Those cases did not harbor any mutation. Distinction of those oncocytic hyperplastic nodules from oncocytic adenomas (Figures 1-2) is not possible based on cytomorphological features or the established criteria of adenoma.

Two additional cases of Hashimoto’s disease presenting oncocytic nodules (Figure 3A-3B and Figure 3C-3D, respectively) in the setting of nodular hyperplasia were retrieved. One of these cases was accompanied by two foci of papillary microcarcinoma.

NGS analysis (Illumina platform) revealed an NRAS mutation in exon 3 in the case of the solitary oncocytic nodule. Once again, a nodule lacking the established features of an adenoma displayed a pathogenic mutation, suggesting a neoplastic process rather than a reactive one, which prompted us to reconsider it as an oncocytic adenoma. No mutation was identified in the cases of Hashimoto’s disease. The characteristics of each case are shown in detail in Table 1.

**Table 1:** Clinicopathological characteristics of examined cases IHC: immunohistochemistry, OA: oncocytic adenoma, PT micro Ca: papillary thyroid microcarcinoma. *Heterogeneous stain, **normal follicular cells also showed positivity.

Case	Sex	Age (yrs)	Histological features	Oncocytic nodule	Presence of other oncocytes	p53 IHC	Cyclin D1 IHC	b-catenin IHC	Ki67 IHC	Pathogenic mutations	Final diagnosis of oncocytic nodule
1	F	40–45	Nodular hyperplasia: a solitary oncocytic nodule	0.8 cm	No	+	+	+	Low	TP53	OA
2	F	45–50	Nodular Hashimoto disease: oncocytic hyperplastic nodule	0.5 cm	Yes	+	+	+	Low	None	Oncocytic hyperplastic nodule
3	F	75–80	Nodular hyperplasia: a solitary oncocytic nodule	0.7 cm	No	+	+**	+	Low	NRAS	OA
4	F	60–65	PT microCa: nodular Hashimoto disease-oncocytic hyperplastic nodule	0.9 cm	Yes (no capsule)	+*	+	+	Low	None	Oncocytic hyperplastic nodule

The above findings indicate that solitary nodules consisting of oncocytes may actually represent adenomas, regardless of the absence of the established criteria to diagnose an adenoma. Taking into account that the morphology of oncocytes is basically the same in hyperplastic nodules and in adenomas, we tried to investigate immunohistochemically if there is any difference in the expression of previously used proteins in oncocytic neoplasms, such as b-catenin [9], Cyclin D1, p53, and Ki67 [10], without any significant result. Immunohistochemical staining for p53 showed positivity in all cases. Moreover, we could not consider p53 protein expression as a reliable marker, taking into account that the surrounding follicular cells also showed variable reactivity. A low proliferation index, as expected in benign lesions, was confirmed in our cases. All immunohistochemical and molecular data for the four tested cases are shown in Table 1.

## Discussion

We present four cases with similar solitary oncocytic lesions. Although they would all be histologically classified as oncocytic hyperplastic nodules, we revealed through molecular analysis that the first two of them carried pathogenic mutations in the p53 and NRAS genes, respectively, suggesting a neoplastic/clonal process.

The distinction between hyperplastic nodules and adenomas is sometimes a diagnostic challenge, occasionally raising controversies in our daily practice. Differential diagnosis between them is not always possible without molecular studies (polyclonal proliferation versus monoclonal neoplasm) [3,4,11]. Features favoring the diagnosis of adenoma include the presence of a well-defined fibrous capsule, architectural and cytological differences from the surrounding gland, signs of compression in the surrounding gland, and closely packed follicles, trabeculae, or solid sheets [4,11,12].

Problems of classification may arise in ambiguous cases with the focal presence of a thin capsule, the absence of any sign of compression, or the presence of a solitary nodule with a distinct morphology lacking the other features of an adenoma as described above. Baloch et al. are proposing an alternative terminology to address this problem, namely “thyroid follicular nodular disease," a term that achieved consensus support [3,4]. The aim is to use a term that will obsolete the need to designate a nodule as "hyperplastic," "neoplastic,” or the contradictory “adenomatous hyperplasia," since this is not always possible with an H&E-stained section. When multifocal benign nodules are present, this term can aid our diagnosis. However, it may cause confusion when it comes to the designation of a solitary nodule as hyperplastic or as an adenoma with the above-described features and, therefore, fails to elucidate the differences in the underlying pathophysiological mechanism. According to the new World Health Organization (WHO) blue book, the distinction between OA and an oncocytic hyperplastic nodule is straightforward only when the tumor is solitary and has a well-defined capsule [4]. When the latter is missing, diagnostic concerns may arise.

Unfortunately, in terms of nodular hyperplasia and OA, the molecular landscape is not fully elucidated [4,13,14], and there is no reliable molecular test or immunohistochemical stain to differentiate them. Although OAs generally behave in a benign fashion, the molecular signature of these lesions suggests a possible malignant phenotype [13,15], and therefore, further investigation might be necessary. It is well known that widely invasive malignancies have a distinct profile, including activation of the PIK3CA-Akt-mTOR and Wnt/b-catenin pathways [5,13,15] and that adenomas cluster with minimally invasive carcinomas [5]. Mutations in p53 and NRAS genes in oncocytic lesions have been previously reported in the literature [14-17]. However, their significance remains unclear since the current results are not definitive in determining clinical behavior and, therefore, management. Nikitski et al. highlight the possible role of the p53 mutation in thyroid carcinogenesis, proposing that TP53 mutant adenomas may represent precursors of thyroid cancer [17]. The use of molecular analysis in our routine to differentiate between OAs and oncocytic hyperplastic nodules is not feasible or practical, and there are no clear-cut OA-specific mutations, enhancing even more the necessity of robust histological criteria. Altogether, the above data highlight the importance of not underdiagnosing an OA as an oncocytic hyperplastic nodule and, furthermore, adopting a common and accurate terminology for oncocytic lesions in our daily practice. Keep in mind that in cases of large OAs, the whole capsule should be histologically examined, and moreover, we need to keep in mind the clinical implications of such a distinction since the follow-up of patients with OAs should be different from the follow-up of patients with nodular hyperplasia.

## Conclusions

Controversial cases with oncocytic nodules do exist in our daily practice. The authors of the new WHO Blue Book clearly state that in some cases, histologic distinction between a clonal OA and a hyperplastic nodule with oncocytic change is not possible. Although our study is limited by the small number of cases included, we were able to identify two cases that would otherwise be diagnosed as oncocytic hyperplastic nodules harboring pathogenic mutations, a feature suggestive of a neoplastic or clonal process rather than a reactive one. Therefore, we emphasize the necessity of the introduction of straightforward guidelines regarding the designation of such lesions. These preliminary data indicate that a solitary oncocytic nodule in a thyroid specimen that lacks the presence of any oncocytes elsewhere demonstrates the neoplastic nature of the lesion and is proposed to be designated as an oncocytic adenoma regardless of the fulfillment of the well-established morphologic criteria of an adenoma (i.e., ambiguous presence of well-defined capsule). Larger studies are needed to elucidate, beyond any doubt, such equivocal cases and propose clear-cut morphologic criteria regarding their more accurate classification.
